# Multi-Agent Deep Reinforcement Learning for Regional Traffic Signal Control Based on Dynamic Weight Decomposition

**DOI:** 10.3390/s26185766

**Published:** 2026-09-11

**Authors:** Peng Shi, Zhenghua Zhang

**Affiliations:** 1 Guangling College, Yangzhou University, Yangzhou 225012, China; 060056@yzu.edu.cn; 2 College of Information and Artificial Intelligence (College of Industrial Software), Yangzhou University, Yangzhou 225012, China

**Keywords:** traffic signal control, multi-agent reinforcement learning, attention mechanism, simulation of urban mobility (SUMO)

## Abstract

Conventional traffic signal control methodologies are deficient in adapting to rapid traffic flow variations and capturing the complex dynamic interactions between intersections within regional road networks. In order to address this specific issue, the present study proposed the Qatten (Q-value Attention Network)-TSC algorithm. The algorithm was constructed on the basis of the dynamic weighted value decomposition principle and was built upon the multi-agent QMIX (Q-value Mixed Network) framework. The model employed a multi-head attention mechanism to effectively fuse individual agent Q-values with global states and individual features to compute global Q-values. Furthermore, the model incorporated multidimensional state information to comprehensively characterize complex traffic networks. Extensive experiments were conducted on small- and large-scale SUMO simulation platforms based on the real road network of Yangzhou. The experimental results demonstrated that in comparison to VDN and QMIX, Qatten-TSC attained average reward increments of 26.4% and 3.46%, correspondingly, in small-scale road networks, and 34.12% and 12.81%, correspondingly, in large-scale road networks. Furthermore, in large-scale scenarios, the average time loss was reduced by 19.28% and 7.15%, respectively, while the average speed increased by 3.00% and 0.87%, respectively. In addition, the baseline algorithm (Qatten) is unstable and poor-performing. The dynamic weighting mechanism is robust and effective, even as the road network complexity increases.

## 1. Introduction

The concept of smart cities is gaining rapid traction worldwide, with intelligent transportation systems emerging as a pivotal component of these urban developments. These systems are designed to address critical issues such as traffic congestion and improve travel efficiency [1]. In its 2023 report, the global transportation organization INRIX observed that, due to traffic congestion, residents of New York, London and Paris lose an average of 101, 99 and 97 h per person per year, respectively. In the United States, the total economic loss caused by traffic congestion is estimated to be 70 billion U.S. dollars. Congestion on urban roads has been shown to generate large amounts of carbon dioxide, as well as wasting fuel and other resources due to the frequent start-and-stop behavior of vehicles [2]. In recent years, many countries are leveraging their existing transportation infrastructure to develop intelligent transportation systems with a view to improving traffic control and management [3].

Intersections represent pivotal nodes within urban road networks, where congestion can markedly impede network-wide traffic efficiency. The optimization of signal control at intersections has been identified as a highly effective measure in the alleviation of urban traffic congestion [4]. The evolution of traffic signal control (TSC) methods commenced in the late 19th century and has since diversified into a range of control approaches. Within this field, traffic signal control based on non-reinforcement learning methods can be categorized into three distinct classifications: timed control [5], sensor-based control [6], and adaptive control [7,8]. The conventional signal control methodologies are predicated on mathematical models and are thus incapable of accommodating the intricate, dynamic and constantly evolving nature of traffic flow [9]. Consequently, they are not well-suited for practical implementation in extensive road networks [10].

The field of reinforcement learning (RL), which does not necessitate prior knowledge or mathematical models of the environment, has been shown to achieve strong performance in large-scale and complex non-linear systems. Consequently, it has emerged as a significant research area within the domain of intelligent transportation [11]. The field of RL-based traffic signal control can be categorized into two distinct classifications: single-point intersection control and multi-point intersection control. In the context of deep reinforcement learning (DRL) applications for single-intersection traffic signal control, the traffic signal controller at a single intersection is frequently regarded as an agent responsible for adjusting signal phases. Variables such as queue length and delay time are utilized as rewards to optimize the signal control strategy. In order to reduce the average waiting time for vehicles at intersections, researchers combined the high-dimensional data processing capabilities of deep learning with the dynamic decision-making advantages of reinforcement learning [12,13]. Multi-intersection control emphasizes dynamic coordination across the road network. However, when using traditional single-agent control methods, independent decision-making at each intersection can lead to mismatched green light sequences. This can cause traffic backlogs that spread. Cooperative multi-agent algorithms can help, but they can be susceptible to failures and computational bottlenecks [14]. In order to address this issue, a fully decentralized framework was introduced, which liberates each agent from the constraints of a central controller, thus enabling it to make decisions and learn based on its own experience [15]. However, the random choices made by agents during exploration are perceived by other agents as unstable dynamics in the environment, leading to uncertainty during training and making it difficult to achieve theoretical convergence [16].

The advent of the Centralized Training and Decentralized Execution (CTDE) architecture has, to a certain degree, addressed the issues identified in both fully centralized and fully decentralized architectures. The prevailing mainstream architecture for collaborative systems, CTDE, exhibits distinct advantages. During the training phase, a central controller employs global information to enhance learning efficiency, while during the execution phase, each agent makes independent decisions, thereby ensuring the system’s scalability and flexibility [17]. Whilst the employment of deep reinforcement learning in the adaptive control of traffic signals has enhanced traffic flow efficiency, regional traffic signal control continues to encounter challenges pertaining to coordination efficiency and complex dynamic interactions. Moreover, the majority of extant research on traffic signal control has been validated on synthetic road networks and lacks support from multi-scale scenario experiments based on real urban road networks. In order to address these challenges, this paper proposed a multi-agent reinforcement learning algorithm. This algorithm was based on dynamic weighted value decomposition. The innovative integration of a multi-head attention mechanism into the CTDE framework enables the coordinated optimization of regional road networks. The main contributions and findings of this study are summarized as follows:

- Enhanced Representation of Dynamic Interactions: by introducing a multi-head attention mechanism into the value decomposition network, Qatten-TSC effectively captures the non-linear and spatio-temporal dependencies among multiple intersections from different representation subspaces.
- Dynamic Weighted Aggregation Strategy: unlike existing attention-based methods that rely on static sum aggregation, Qatten-TSC utilizes a global state-dependent dynamic weighting mechanism. This strategy adaptively adjusts the importance of different attention heads, ensuring that the agent focuses on critical collaborative features while filtering out noise, thereby significantly improving the accuracy of value decomposition.
- Validation Experiments Based on Real-World Traffic Conditions: extensive experiments conducted on SUMO simulation platforms, using both small-scale and large-scale road networks modeled after real-world scenarios in Yangzhou, Jiangsu, China, demonstrate the efficacy of the proposed approach.

The rest of this paper is organized as follows: Section 3 presents the Qatten-TSC model for the control of traffic signals at multiple intersections. Section 4 introduces the experimental validation and results analysis. Section 5 discusses the results and identifies future research directions. Section 6 summarizes the key findings, highlights the study’s significance, and puts forward directions for future research.

## 2. Related Works

In multi-agent reinforcement learning (MARL), the joint action space of a multi-agent system exhibits exponential growth with the number of agents, and partial observability gives rise to environmental non-stationarity. In response to this challenge, the value factorization technique was developed, whose core lies in decomposing the global action-value function into individual action-value functions for each agent under the CTDE paradigm. This approach ensures consistency between individual and global optimality, as outlined in the Individual-Global-Max (IGM) principle. Sunehag, P. et al. proposed the Value Decomposition Network (VDN), a seminal work in this field. The global Q-value was decomposed directly into the sum of the individual Q-values of each agent via linear summation. This approach featured a simple structure and was straightforward to implement [18]. However, this mechanism is incapable of modeling complex nonlinear dependencies between agents, resulting in limited expressiveness for collaborative multi-agent tasks involving strong interactions. To address this issue, Rashid, T. et al. proposed the QMIX (Q-value Mixing Network) algorithm. This development resulted in the establishment of a hybrid network that incorporated individual Q-values into a global Q-value, thereby facilitating nonlinear integration. Constraints were imposed on the model to ensure compliance with the IGM principle. This intervention led to a marked enhancement in performance levels when undertaking complex collaborative tasks [19]. However, these constraints impose a structural limitation on the representational capacity of the joint value function, making it difficult to characterize global value functions with non-monotonic characteristics. To address this, Rashid, T. et al. proposed Weighted QMIX (WQMIX), which expands the representation range of the monotonic value function decomposition by dynamically adjusting the weights of each agent’s Q-values. This approach alleviated the suboptimal issues caused by the monotonicity constraint [20]. However, the weighting mechanism still relies on preset heuristic rules and lacks flexibility in adapting to state changes. Wang, J. et al. presented the QPLEX (Duplex dueling multi-agent q-learning) algorithm, which employs a ‘Duplex Dueling’ architecture for the purpose of decomposing the joint action-value function into value and advantage terms. This approach does not rely on monotonicity constraints and fully utilizes the expressive power of the IGM function family. It is currently considered to be one of the most expressive value decomposition frameworks [21]. However, all of the methods described above rely on fixed or heuristic aggregation strategies that lack adaptive, state-dependent weighting over interaction patterns. The inability to dynamically adjust the distribution of attention weights based on the current global state renders these systems challenging to adapt to rapidly changing interactions between intersections in a traffic system over time.

In order to enhance the ability to model dynamic interactions among agents during value decomposition, attention mechanisms have been introduced into the field of MARL. Yang, Y. et al. proposed the Qatten algorithm, which utilizes a multi-head attention mechanism to represent the global Q-value as a linearly weighted combination of the individual Q-values of each agent. This approach theoretically guarantees the IGM property and can capture interaction patterns among agents from different subspaces, offering greater expressive power than QMIX’s single hybrid network [22]. Zhang, H. et al. proposed the Qrainbow algorithm for heterogeneous multi-robot collaboration tasks. This pioneering algorithm also employs Qatten’s multi-head attention blending network. The efficacy of the attention blending mechanism in scenarios involving heterogeneous agents was demonstrated through the combination of techniques such as dual networks and priority experience replay [23]. However, it should be noted that the aforementioned methods generally employ an equally weighted summation when aggregating multi-head attention outputs. This approach disregards the varying levels of importance attributed to diverse interaction patterns within dynamic environments. This phenomenon results in the attenuation of key features and the introduction of redundant information, leading to inaccuracies in credit allocation in road network control tasks where traffic flow conditions change rapidly. Consequently, this limits the performance ceiling of such methods in complex scenarios.

Graph neural networks (GNNs) are extensively employed in traffic network topology modeling, a field in which their capacity to process data in non-Euclidean spaces is particularly advantageous. Wang, T. et al. proposed MGMQ, an algorithm for the control of traffic signals at scale that is based on multi-layer graph mask Q-learning. The proposed methodology involves the division of the traffic environment into an intersection layer and a road network layer. The intersection layer is generated using GAT, with an improved GraphSAGE being used to aggregate information and generate intersection state embeddings that incorporate geometric and spatial features. The model attains zero-shot transfer through the utilization of action masking and parameter sharing [24]. Zhou, T. et al. proposed GMIX, a graph-based spatial–temporal multi-agent reinforcement learning framework that integrates a spatial–temporal graph attention network (ST-GAT) with a value decomposition algorithm termed Graph-QMIX. By replacing the conventional mixing network with a graph attention-based one, GMIX explicitly models the structural interactions among agents while satisfying the monotonicity constraint, and demonstrates superior performance in dynamic electric vehicle dispatching tasks [25]. However, GMIX is designed for vehicle routing and dispatching rather than traffic signal control, and its graph-based mixing still enforces the monotonic assumption, which limits the expressiveness of the joint value function in the same manner as QMIX. Moreover, its aggregation weights are derived from adjacency-level attention coefficients rather than being adaptively conditioned on the global traffic state. Li, X. et al. proposed the concept of multi-hop upstream pressure, extending the definition of pressure to more distant upstream links based on the principles of Markov chain theory. This development enables agents to perceive a broader spatial context. This approach led to a substantial reduction in network latency across both synthetic road networks and the real-world Toronto road network tests [26]. However, the incorporation of multi-hop information also resulted in an escalation in learning complexity and necessitated an increase in computational overhead.

In summary, extant value decomposition methods still have significant shortcomings in terms of contribution allocation: It is evident that both VDN and QMIX possess fixed aggregation weight structures, which hinders the capacity to accurately mirror real-time fluctuations in traffic conditions. The weighting strategies employed by WQMIX and QPLEX are contingent on heuristic rules or are exclusively applicable to individual advantage representations. Consequently, these strategies fail to facilitate adaptive adjustment based on the global state during the joint aggregation stage. Existing methods that are predicated on attention mechanisms predominantly utilize equal-weight summation for multi-head outputs. This limitation impedes their ability to adapt to the time-varying nature of contribution levels across diverse interaction modes. Consequently, the challenge of adaptively allocating aggregation weights for multi-head attention, whilst preserving the IGM property and linear complexity, remains a significant obstacle in the realm of regional traffic signal control. The present paper is concerned with precisely this issue.

## 3. Proposed Methods

### 3.1. Regional Traffic Signal Control Algorithm Based on Dynamic Weighted Value Decomposition

**Figure 1:**
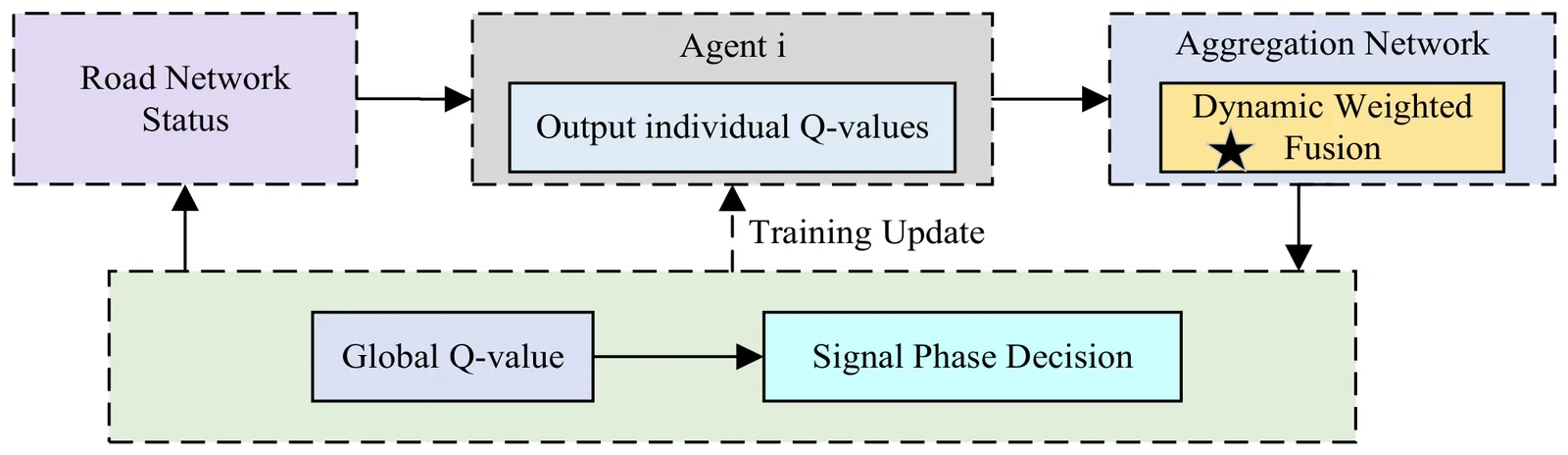
Overall Architecture Diagram of the Qatten-TSC Algorithm.

This paper proposed the Qatten-TSC algorithm as a refinement of value decomposition-based multi-agent reinforcement learning frameworks. At the framework level, this paper adopted well-established designs from prior research: multi-agent modeling following the CTDE paradigm and the global Q-value decomposition approach proposed by VDN [18]; the neural network architecture for each agent was adapted from QMIX [19]; and the multi-head attention decomposition framework was adapted from Qatten [22]. The paper’s pioneering contribution lies in its dynamic weighting mechanism, which was incorporated into the attention aggregation stage. In contrast to existing methods that employ equal-weight summation during aggregation, this mechanism adapted to the time-varying characteristics of traffic flow. The overall framework and workflow of Qatten-TSC are illustrated in Figure 1. The regional traffic network environment provides the global state, local observations for each agent, and rewards. At each intersection, agents generate individual Q-values based on their local observations using individual Q-networks and select phase actions. The aggregation network fuses individual Q-values through multi-head attention and generates a global Q-value by adaptively assigning weights to each head based on the global state using the dynamic head weighting mechanism innovated in this paper (marked with an asterisk in the figure). The training module stores interaction data in an experience replay pool and utilizes a target network and parameter synchronisation mechanism to stabilize the training process.

#### 3.1.1. Value Decomposition Multi-Agent Reinforcement Learning Algorithm

VDN, an early value decomposition algorithm, learns by decomposing the global Q-value $Q_{total}\left(s_{t},u_{t}\right)$ into the sum of the individual Q-values of each agent. The linear summation mechanism of VDN is shown in Equation (1) [18].

(1) $$Q_{total}\left(s_{t},u_{t}\right)=-\sum_{i=1}^{N}Q_{i}\left(\tau_{t}^{i},a_{t}^{i}\right)$$

Here, $s_{t}$ denotes the global state, $u_{t}=\left(a_{t}^{1},\ldots ,a_{t}^{i},\ldots ,a_{t}^{N}\right)$ signifies the joint action, $a_{t}^{i}$ represents the individual action of agent *i*, and $\tau_{t}^{i}$ denotes the action history of agent *i*. *N* is the number of agents. However, the direct summation mechanism employed results in the individual Q-values $Q_{i}\left(\tau_{t}^{i},a_{t}^{i}\right)$ of each agent being simply added together following independent training. This approach is found to be deficient in its modeling of the nonlinear dependencies among agents, which consequently results in sub-optimal performance in complex collaborative tasks.

QMIX employs a nonlinear value decomposition based on VDN, utilizing a hybrid network to integrate the individual Q-values of each agent [27,28]. Furthermore, it designs hypernetwork parameters based on the global state $s_{t}$. While satisfying monotonicity constraints such as those in Equation (2) [19], it extends the linear summation to a state-dependent nonlinear combination; the extended form is shown in Equation (3). This ensures that the optimal solution for individual strategies aligns with the globally optimal decision while also enabling the modeling of complex dynamic interactions among agents, thereby enhancing adaptability in collaborative tasks.

(2) $$\frac{\partial Q_{total}}{\partial Q_{t}}\geq 0,\forall i$$

(3) $$Q_{total}\left(s_{t},u_{t}\right)=f_{min}\left(s_{t},Q_{1}\left(\tau_{t}^{i},a_{t}^{i}\right),\ldots ,Q_{N}\left(\tau_{t}^{N},a_{t}^{N}\right)\right)$$

By leveraging the monotonicity constraints delineated above, QMIX guarantees that the optimal solution for the global Q-value of $Q_{total}\left(s_{t},u_{t}\right)$ is indisputably equivalent to the optimal amalgamation of actions for each agent’s individual Q-values, as demonstrated in Equation (4) [19].

(4) $$\underset{u_{t}}{\arg\max}\;Q_{total}\left(s_{t},u_{t}\right)=\left[\begin{array}{ccc} \underset{a_{t}^{1}}{\arg\max}\;Q_{1}\left(\tau_{t}^{1},a_{t}^{1}\right), & \ldots , & \underset{a_{t}^{N}}{\arg\max}\;Q_{N}\left(\tau_{t}^{N},a_{t}^{N}\right) \end{array}\right]$$

#### 3.1.2. Qatten-TSC Model for Multi-Intersection Traffic Signal Control

**Figure 2:**
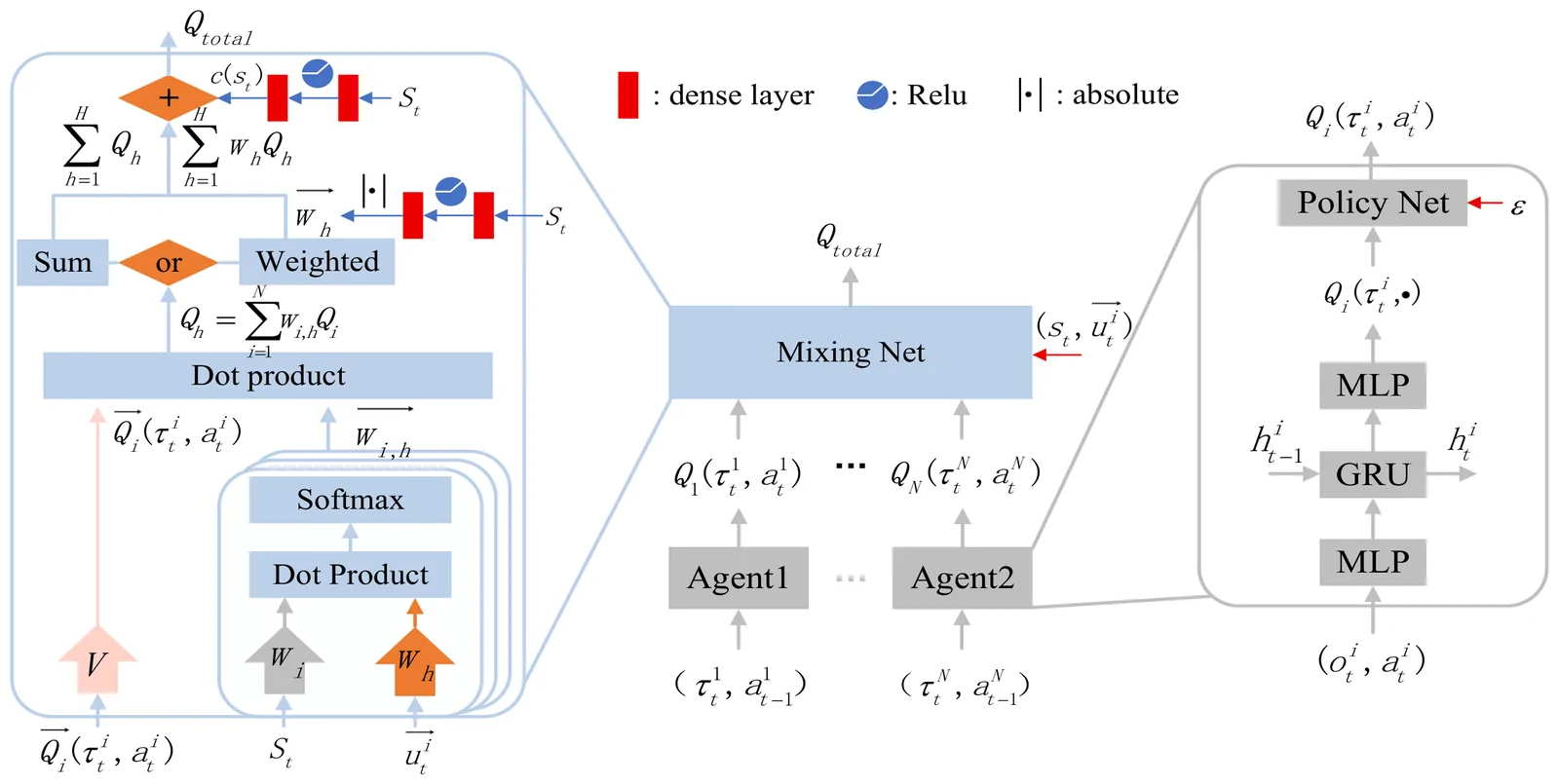
Network Architecture of the Qatten Algorithm (Adapted from Figure 1 in Reference [22]).

This paper proposed Qatten-TSC, a regional traffic signal control algorithm based on dynamic weighted value decomposition, building on QMIX. This algorithm employed a multi-head attention mechanism to capture the complex interactions among intersections across different subspaces, thereby effectively decomposing the global Q-value into individual Q-values for each agent, which ensured the expressive power of the value decomposition. The Qatten network architecture is illustrated in Figure 2. In the right-hand side of the architecture, there is the recurrent deep Q-network for each agent, which receives the action-observation history record $\tau_{i}$ (last hidden states $h_{t-1}^{i}$, current local observations $o_{t}^{i}$ and last action $a_{t-1}^{i}$). This information is subsequently processed through a multi-layer perceptron (MLP), a gated recurrent unit (GRU) layer, and a subsequent MLP, culminating in the generation of the agent’s Q-value. On the left side of the architecture, the aggregation network employs a multi-head attention mechanism to fuse the individual Q-values of all agents with the global state $s_{t}$ and individual features $u_{t}^{i}$ to compute the global Q-value $Q_{total}\left(s_{t},u_{t}\right)$.

The global Q-value can be calculated using either the direct summation (Sum) method or the weighted summation (Weighted) method. As demonstrated in Equation (5), the direct summation method for calculating $Q_{total}$ is achieved by equally summing the outputs of each attention head [22].

(5) $$Q_{total}\left(s_{t},u_{t}\right)=c\left(s_{t}\right)+\sum_{h=1}^{H}\sum_{i=1}^{N}w_{i,h}Q_{i}\left(\tau_{t}^{i},a_{t}^{i}\right)$$

The weighted sum calculation of $Q_{total}$, as demonstrated in Equation (6), dynamically adjusts the importance of each head using the head weight $w_{h}$ to enhance adaptability to complex tasks [22]. It was evident that the Qatten-TSC algorithm delineated in this study was implemented in accordance with the weighted sum method.

(6) $$Q_{total}\left(s_{t},u_{t}\right)=c\left(s_{t}\right)+\sum_{h=1}^{H}w_{h}\sum_{i=1}^{N}w_{i,h}Q_{i}\left(\tau_{t}^{i},a_{t}^{i}\right)$$

As illustrated in Equations (5) and (6), the symbol $w_{i,h}$ denotes the attention weight of the *h*-th head of agent *i*, and the symbol $c(s_{t})$ signifies a state-dependent constant term. *H* is the number of attention heads.

Dynamic weights are incorporated into the aggregation process as a linear combination of individual Q-values. This mechanism preserves the IGM properties while introducing only N×H additional scalar parameters. Furthermore, the computational complexity of the aggregation process increases linearly with the number of agents.

In summary, the present study successfully achieved an effective decomposition of the global Q-value into individual Q-values by means of an aggregation network. Specifically, the aggregation network employed a multi-head attention mechanism to extract deep interaction features among agents at each intersection, combining these with the global state $s_{t}$ to generate dynamic weights $w_{h}$. The temporal difference error of the global Q-value was minimized, and gradient signals were fed back through the aggregation network to the individual networks of each agent. This mechanism compelled individual Q-values not only to fit local action values but also to accurately reflect their contribution to global coordination. This ensured the expressive power and accuracy of the value decomposition model when handling complex coupling relationships within the road network.

#### 3.1.3. Training Process for the Algorithm

**Figure 3:**
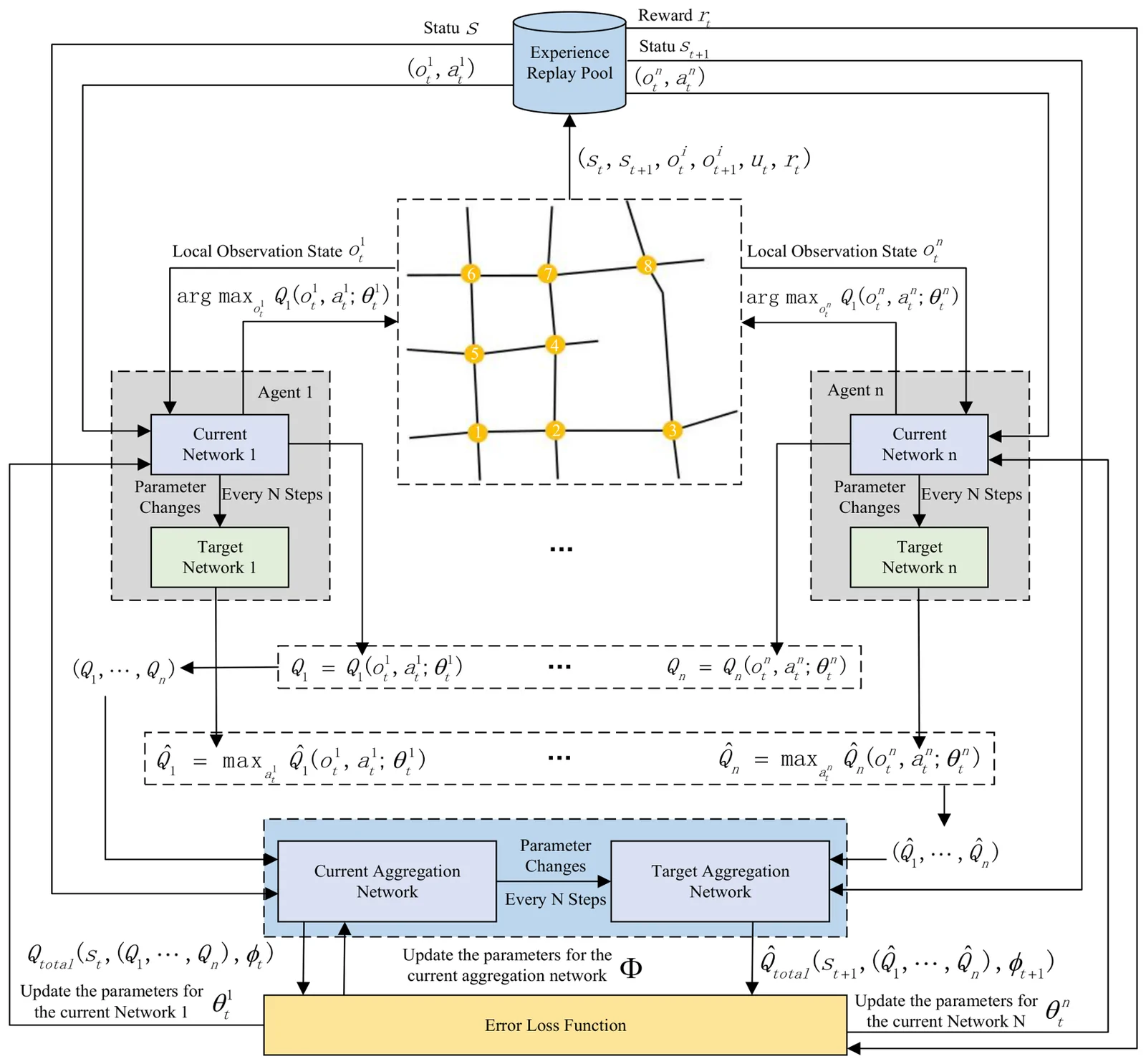
Training Map for Regional Traffic Signal Control Based on the Qatten-TSC Algorithm.

Figure 3 illustrates the training process of the model for coordinated traffic signal control at multiple intersections in a road network based on the Qatten-TSC algorithm.

In the training process for regional traffic signal control based on the Qatten-TSC algorithm, all agents and aggregation networks adopt a dual-network architecture consisting of a current network and a target network. The target network synchronizes parameters from the current network through *N* updates to reduce the bias in Q-value estimation. Specifically, in each training iteration, the agent interacts with the intersection in the area using the current network, generates an individual state-action Q-value $Q_{i}\left(\tau_{t}^{i},a_{t}^{i}\right)$ based on local observations, and selects a single action $a_{t}^{i}$ that maximizes the Q-value to adjust the traffic light phase, thereby forming a joint action set $u_{t}$. The environment subsequently transitions to the subsequent global state $s_{t+1}$, and the global reward $r_{t}$ is returned. Concurrently, the interaction data sextuplet $\left(s_{t},s_{t+1},o_{t}^{i},o_{t+1}^{i},u_{t},r_{t}\right)$ is stored in the experience replay pool. When the experience replay pool is full, a certain batch of data is randomly sampled. The current network outputs the individual Q-values $Q_{cur}=\left(Q_{1}\left(\tau_{t}^{1},a_{t}^{1}\right),\ldots ,Q_{N}\left(\tau_{t}^{N},a_{t}^{N}\right)\right)$ for each agent, and the target network outputs the target Q-value $Q_{target}$. The current aggregation network then combines the global state $s_{t}$, $Q_{cur}$, and the locally observed $\left\{o_{t}^{1},\ldots ,o_{t}^{N}\right\}$ to generate the current global estimate $Q_{total}\left(s_{t},u_{t}\right)$. The target aggregation network calculates the global target value $Q_{total}\left(s_{t+1},u_{t+1}\right)$ based on the next state $s_{t+1}$ and $Q_{target}$. Subsequently, the error is calculated by combining the reward $r_{t}$ with the loss function. In addition, the current network parameters $\theta_{i}$ and the aggregation network parameters Φ are updated via back propagation. Concurrently, the prevailing network parameters are synchronized to the target network at regular intervals of *N* steps, thereby stabilizing the training process and optimizing the multi-intersection cooperative control strategy.

### 3.2. Agent Design

In the context of the coordinated management of a regional road network, multiple intersections function as agents and collectively engage in decision-making processes. In the context of signal control in a regional road network, it is imperative to consider factors such as global observation information and coordinated actions. This paper proposed a definition of the state space, action space, and reward space of the multi-agent algorithm, as outlined below.

#### 3.2.1. State Setting

Changes in the road network state are essentially a continuous, dynamic process; continuously and accurately modeling the micro-level dynamics of traffic flow would cause computational complexity to rise sharply. The Discrete Traffic State Encoding (DTSE) method introduces a discrete modeling framework that represents complex traffic states by constructing multidimensional state matrices [29,30]. In accordance with the Discrete Traffic State Encoding framework, the state design of multi-agent algorithms is required to encompass both the local characteristics of individual intersections and the global dynamics of the entire regional road network. Consequently, the system state comprises both the local observation state *o* and the global state $S_{t}$. The global state $S_{t}$ is defined as the aggregation of the local observed states *o* of all intersections within the regional road network at time step *t*, and is used to reflect the overall traffic conditions at these intersections. In the context of multi-agent regional road network control, it is imperative that local states consider a multitude of road information types to facilitate the attainment of coordinated global control [31,32]. The global state $S_{t}$ is defined as a multidimensional vector that consolidates the local states of all *n* intersections, as illustrated in Equation (7).

(7) $$s_{t}=\left[\begin{array}{c} o^{1}\\ o^{2}\\ \vdots \\ o^{i}\\ \vdots \\ o^{n} \end{array}\right],o^{i}=\left[\begin{array}{cccc} P^{i}, & T_{flag}^{i}, & D^{i}, & Q^{i} \end{array}\right]$$

where $o^{i}$ denotes the local observation state at the *i*-th intersection, encompassing its green light phase $P^{i}$, the minimum green light duration flag $T_{flag}^{i}$, lane density $D^{i}$, and lane queue length $Q^{i}$. The green light phase at the *i*-th intersection $P^{i}$ is represented by a one-hot encoded vector, which indicates the current traffic signal state at the intersection. The formula is shown in (8).

(8) $$P^{i}=\left[\begin{array}{cccc} p_{1}^{i}, & p_{2}^{i},\cdots , & p_{j}^{i},\cdots , & p_{N}^{i} \end{array}\right]$$

where *N* denotes the number of traffic light phases, $P_{j}^{i}=1$ indicates that the *j*-th phase at the *i*-th intersection is currently green, and $P_{j}^{i}=0$ indicates that the *j*-th phase at the *i*-th intersection is not green.

The minimum green light duration Flag $P_{flag}$ is a binary flag that is utilized to prevent excessive phase changes and ensure the stability of traffic flow. The definition of this phenomenon is as follows: the duration $t_{green}$ of the current green light phase is defined as being less than the sum of the minimum green light duration $T_{min}$ and the yellow light duration $T_{yellow}$. When the condition in Equation (3) is satisfied, $T_{flag}=1$; otherwise, $T_{flag}=0$.

(9) $$t_{green}<T_{min}+T_{yellow}$$

The lane density at the *i*-th intersection $D^{i}$, representing the density of traffic flow in each lane, is a vector of length *L*, as demonstrated in Equation (4).

(10) $$D^{i}=\left[\begin{array}{cccc} d_{1}^{i}, & d_{2}^{i},\cdots , & d_{j}^{i},\cdots , & d_{L}^{i} \end{array}\right]$$

where *L* denotes the number of lanes that are subject to control at the intersection, and $d_{j}^{i}$ is defined as the lane density of the *j*-th lane at the *i*-th intersection, calculated as the ratio of traffic volume to lane length.

The lane queue length at the *i*-th intersection $Q^{i}$, representing the number of vehicles in the queue, is a vector of length *L*, as demonstrated in Equation (11).

(11) $$Q^{i}=\left[\begin{array}{cccc} q_{1}^{i}, & q_{2}^{i},\cdots , & q_{j}^{i},\cdots , & q_{L}^{i} \end{array}\right]$$

where $q_{j}^{i}$ represents the number of vehicles queued in lane *j* at the *i*-th intersection.

#### 3.2.2. Action Setting

**Figure 4:**
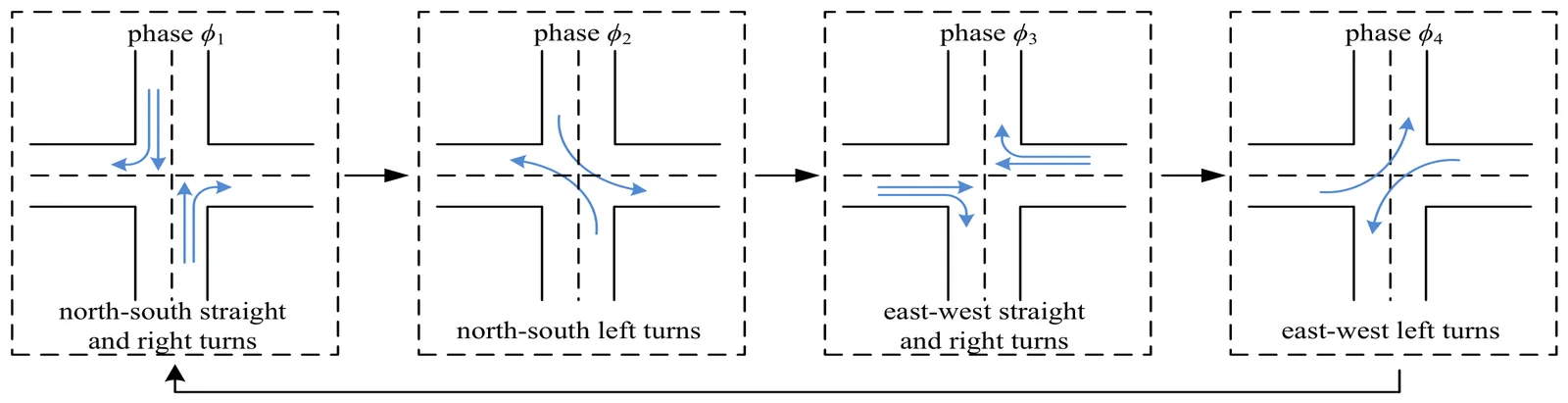
Fixed Phase Sequence Scheme.

Within the domain of traffic signal control, control strategies frequently prioritize phase sequence and phase cycle. Phase sequence dictates the sequence in which traffic flows, whereas phase cycle determines the total duration of a signal cycle and the allocation of time among the phases. In order to ensure adaptability of traffic flow whilst avoiding the risk of driver confusion caused by sudden changes in phase sequence, this paper adopted an adaptive control scheme with a fixed phase sequence but no fixed cycle. With regard to the fixed phase sequence, the four phase schemes depicted in Figure 4 are cycled through in the following sequence: north–south through traffic and right turns, north–south left turns, east–west through traffic and right turns, and east–west left turns.

It is evident that, on the basis of the aforementioned fixed phase sequence, this paper adopted a control strategy that is devoid of a fixed cycle. The agent, in a dynamic manner, adjusts the duration of the green light for each phase by determining the timing of phase transitions, thereby optimizing traffic signal timing. In order to circumvent congestion caused by unduly brief green light durations or superfluous waiting engendered by unduly protracted durations, the green light duration for each phase is constrained between a minimum green light duration of $T_{min}=10$ s and a maximum green light duration of $T_{max}=60$ s, that is, $t_{green}\in \left[T_{min},T_{max}\right]$.

In terms of the implementation of the algorithm, the action space resulting from the combined actions of *n* agents is shown in Equation (12).

(12) $$U_{t}=\left[\begin{array}{c} A^{1}\\ A^{2}\\ \vdots \\ A^{i}\\ \vdots \\ A^{n} \end{array}\right],A^{i}=\left[\begin{array}{cccc} EWL, & EWS, & NSL, & NSS \end{array}\right]$$

At each discrete time step *t*, agent *i* selects an action $a_{t}^{i}$ from its designated action space $A^{i}$, thereby defining its individual action. The individual actions of all agents are then combined into a joint action $u_{t}$, as demonstrated in Equation (13).

(13) $$u_{t}=\left[\begin{array}{cccc} a_{t}^{1}, & a_{t}^{2},\cdots , & a_{t}^{i},\cdots , & a_{t}^{n} \end{array}\right]$$

where $a_{t}^{i}$ denotes the phase selected by the *i*-th agent at time *t*. For instance, $a_{t}^{i}=EWL$ signifies that the agent selected the left-turn phase for east–west traffic at the first intersection. Accordingly, *EWS* signifies east–west straight and right turns, *NSL* denotes north–south left turns, and *NSS* represents north–south straight and right turns.

It is important to note that, although the agent’s actions formally appear as phase selections, the environment strictly limits phase transitions to a fixed phase sequence. In other words, the agent can only transition from the current phase to the next phase in the fixed sequence; skipping phases or selecting arbitrary phases is not permitted. The control problem addressed in this paper is therefore one of adaptive control with a fixed phase sequence and variable period. The phase sequence is guaranteed by structural constraints of the environment, and the agent actually optimizes the green light duration for each phase. This is achieved indirectly by determining the green light duration for that phase by selecting the number of consecutive decision steps to maintain the current phase.

The green light duration constraint is enforced at the environmental level rather than relying on the agent’s voluntary compliance: if the green light duration $t_{green}$ of the current phase is less than $T_{min}=10$ s, the environment disregards the agent’s phase-switching action and forces the current phase to continue. The minimum green light time flag $T_{flag}$ in the state space simultaneously indicates this constrained state, thus assisting the agent in learning the appropriate timing for phase switching. When $T_{flag}$ reaches $T_{max}=60$ s, the environment forcibly ends the current phase. Furthermore, upon the conclusion of the green light phase, the system automatically introduces a 3-s yellow light period to ensure the clearance of conflicting traffic from the intersection, thereby facilitating a safe signal transition. This yellow light period is automatically initiated by the environment and does not utilize any of the agent’s action decision steps. It is evident that conflicts between adjacent phases are resolved via yellow lights and that phase transitions are strictly confined to a fixed sequence. Consequently, it is evident that no illegal or conflicting signal changes occur. Furthermore, it is ensured that the green light duration for each phase always falls within the $\left[T_{min},T_{max}\right]$ interval.

#### 3.2.3. Reward Setting

In the context of signal control algorithms for single-intersection systems, the reward space is conventionally delineated as the alteration in queue length or cumulative delay time between proximate time increments. However, in regional road networks comprising multiple intersections, the performance of a single intersection does not adequately reflect the overall traffic conditions. The primary objective of regional traffic signal control is to alleviate congestion in the road network and enhance traffic efficiency. Queue length is the most direct and intuitive physical metric for measuring the level of congestion at an intersection. The definition of the reward as the sum of negative queue lengths mathematically establishes a strict equivalence between minimizing the reward and minimizing the queue length. This consistent objective function guides the agent to explicitly prioritize actions that effectively clear waiting vehicles during the decision-making process, thereby preventing the strategy from deviating from the essence of optimization. To this end, the reward for each agent *i* was defined as the negative sum of the number of vehicles queued in all lanes at the intersections under its control, as illustrated in Equation (14).

(14) $$r_{t}^{i}=-\sum_{j=1}^{N_{i}}L_{j}^{i}$$

where $N_{i}$ signifies the number of lanes governed by the *i*-th agent, while $L_{j}^{i}$ denotes the quantity of vehicles queued in lane *j* at the *i*-th intersection.

For regional coordination, this algorithm implements the CTDE framework. The global reward $r_{t}$ is the sum of the reward components for all agents in the region, represented as the negative sum of the queue lengths across all lanes, as formulated in Equation (15).

(15) $$r_{t}=-\sum_{i=1}^{N}r_{t}^{i}$$

where *N* is the number of agents. Concurrently, the utilization of the global state $s_{t}$, which encompassed comprehensive density and queuing data, enabled the attention mechanism to discern intricate features such as downstream congestion and the propagation of blockages. Consequently, this ensured that the algorithm was capable of attaining global cooperative optimization.

## 4. Experiment and Analysis

### 4.1. Road Network Modeling

**Figure 5:**
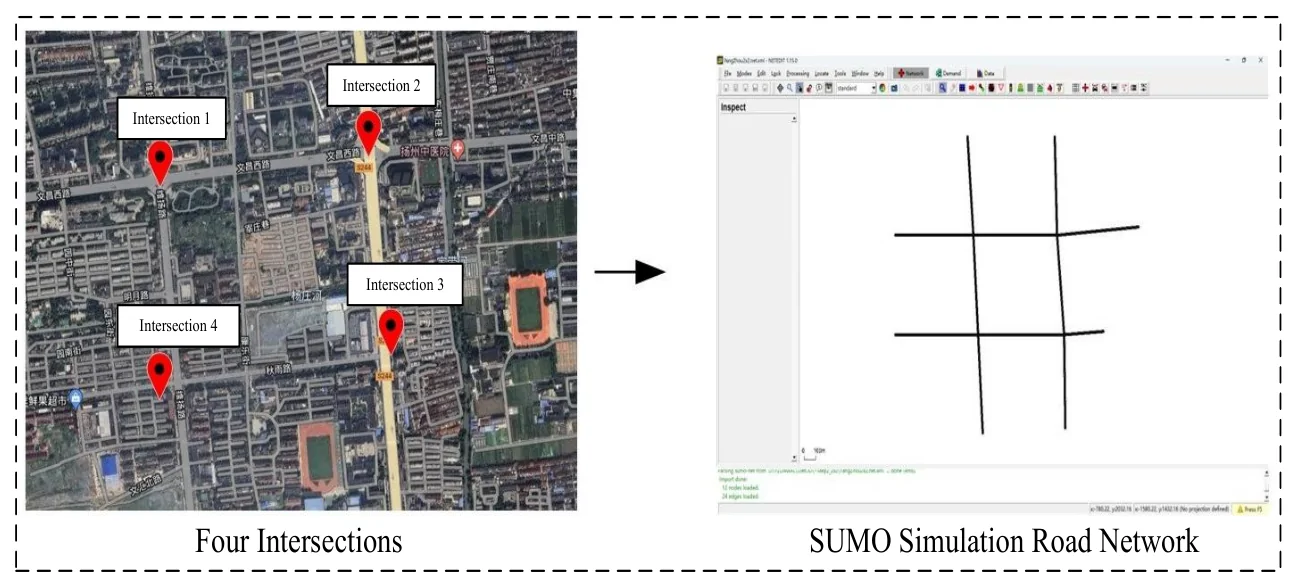
Small-Scale Road Network Model in Yangzhou.

**Figure 6:**
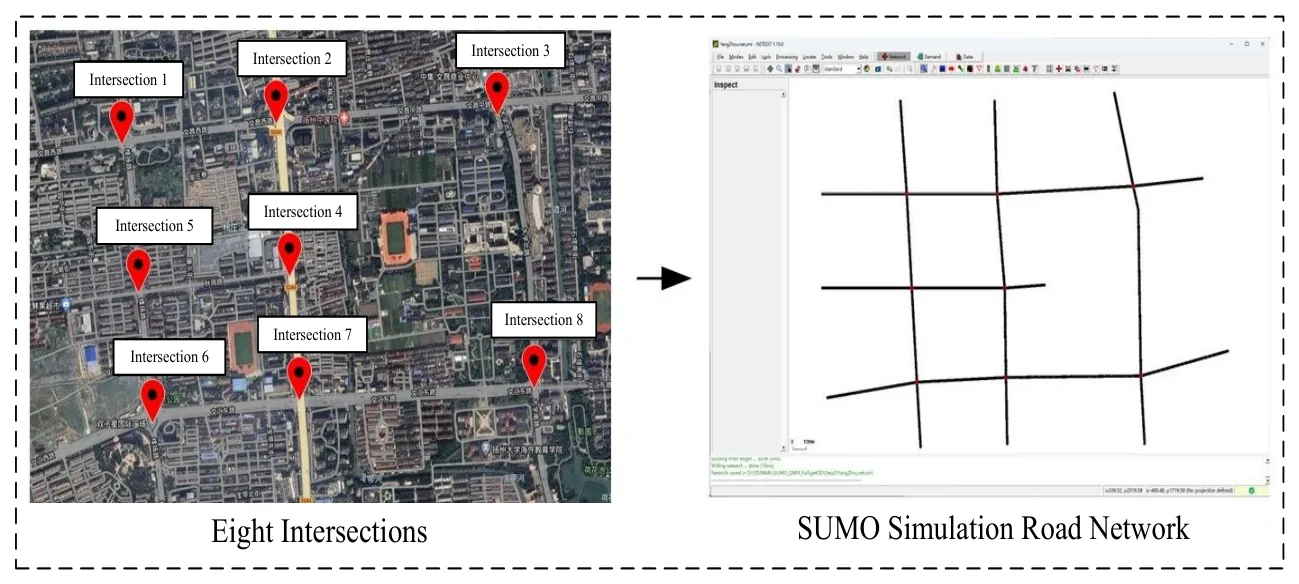
Large-Scale Regional Road Network Model in Yangzhou.

In order to verify the performance of the control algorithm in a complex road network, the SUMO platform’s net-edit tool was used to generate a .net.xml road network file based on the road network of Yangzhou City, Jiangsu Province, China. A comprehensive dynamic traffic simulation scenario was subsequently devised by establishing a connection between the specified file and the .rou.xml traffic flow file through the utilization of a traffic simulation configuration file. This paper presents a road network model for Yangzhou, incorporating two distinct scale scenarios. The initial small-scale road network encompasses a network of four intersections, namely the convergence of Wenchang Road, Weiyang Road, Yangtze River Road, and Qiuyu Road, as depicted in Figure 5. The large-scale regional road network extend to a total of eight intersections, which are formed by the convergence of Wenchang Road, Qiuyu Road, Wenhui Road, Weiyang Road, Yangtze River Road and University Road, as demonstrated in Figure 6. In both types of scenarios, each intersection was equipped with entry and exit lanes in the four cardinal directions (north, south, east and west), with each lane comprising three entry and three exit lanes in each direction. This enabled high-precision dynamic simulation modeling through standardized lane configurations.

**Figure 7:**
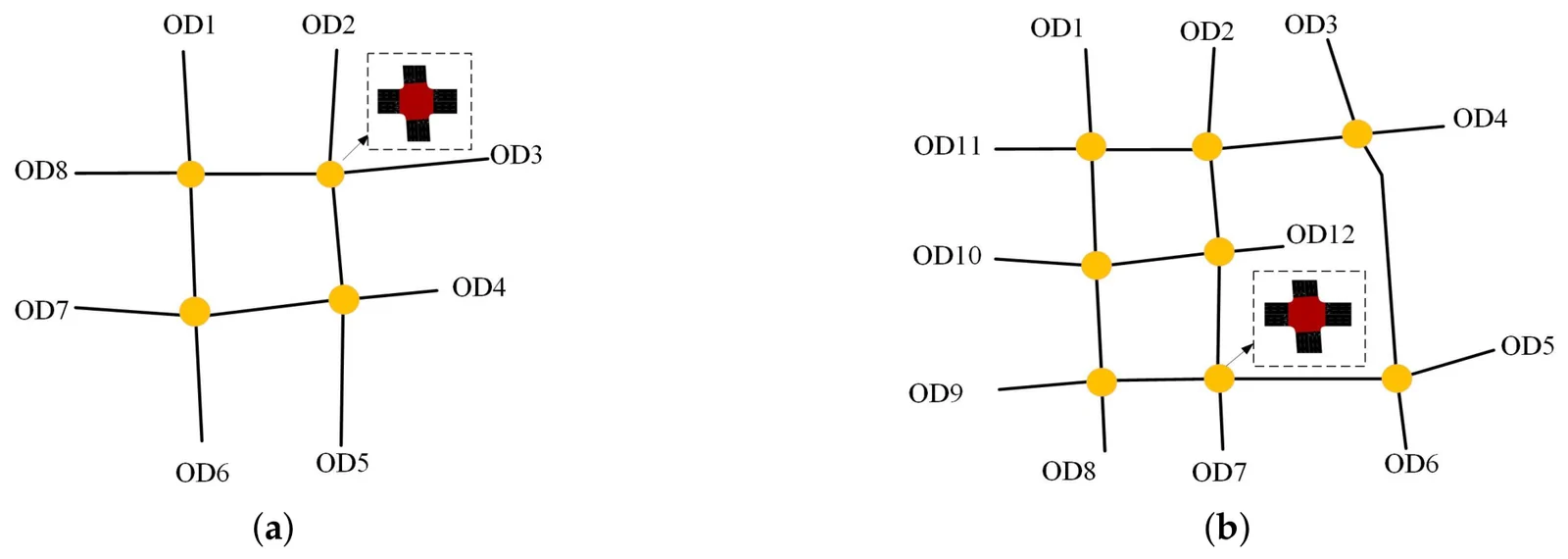
Road network origin–destination matrix. Panel (**a**) shows the OD matrix for a small-scale road network. Panel (**b**) shows the OD matrix for a large-scale regional road network.

Following the completion of the modeling of the Yangzhou road network, it was necessary to inject dynamic traffic flows using an OD matrix in order to construct a complete simulation scenario. As illustrated in Figure 7a, the small-scale road network was deployed, incorporating eight sets of origin–destination (OD) nodes at four intersections, thereby encompassing the entry and exit lanes in all directions. As illustrated in Figure 7b, the large-scale regional road network was deployed, incorporating 12 OD nodes dispersed across 8 intersections. It is imperative to note that all OD nodes were configured in accordance with the actual traffic flow directions. This ensured the accurate simulation of vehicle entry and exit on each lane.

Table 1 and Table 2 present the origin–destination (OD) traffic volume data for incoming lanes in all directions over a one-hour period for small-scale and large-scale regional road networks, respectively. The demand rate was obtained by dividing the OD volume by the simulation duration of 3600 s, i.e., approximately 0.06–0.12 veh/s for each OD pair. Turning percentages were set according to the directions of the OD nodes, with approximately 60% through, 20% left-turn, and 20% right-turn movements at each approach. These data serve as the foundational input for vehicle route generation and are converted into the standard route file (.rou.xml) using the XML conversion tool provided by SUMO, providing accurate traffic demand inputs for the simulation comparison experiments involving road networks of different scales.

The simulation settings are as follows: All vehicles are the default passenger car type of SUMO (vType “passenger”, length 5 m, maximum speed 50 km/h). The lane saturation flow, implicitly determined by the car-following parameters, is approximately 1900–2000 veh/h/lane with the default Krauss parameters, consistent with typical urban values. The default Krauss car-following model (accel=2.6 m/s^2^, decel=4.5 m/s^2^, σ=0.5, τ=1.0 s) and the default LC2013 lane-change model are adopted. Each simulation includes a 600 s warm-up period, and five independent random seeds are used, with results reported as means ± standard deviations over the last 50 training episodes. The Fixed-Time baseline adopts an equal four-phase timing scheme (30 s green plus 3 s yellow per phase) under the same signal environment as the RL methods (i.e., the same fixed phase sequence and yellow transition rules), ensuring that the comparison isolates the effect of the control policy.

### 4.2. Evaluation Indicators

In order to evaluate the performance of the Qatten-TSC algorithm in the context of regional traffic signal control, an examination was conducted from two distinct perspectives: total reward and traffic efficiency. Initially, with regard to the evaluation of the total reward, an evaluation of the overall performance of Qatten-TSC was conducted from the standpoint of reinforcement learning; a higher reward value was indicative of enhanced outcomes with respect to the optimization of traffic signal control. Secondly, with regard to the assessment of traffic efficiency, the metrics employed were based on an analysis of traffic conditions, with the utilization of indicators such as the cumulative length of queues, the mean travel speed, and the mean time loss. In the context of simulation experiments conducted on regional road networks, the minimization of lane queue lengths was designated as the primary optimization objective. A negative reward mechanism was implemented to ensure the maximization of the average reward. Utilizing a dual-criterion approach founded upon mean travel speed and mean time loss, the applicability of the Qatten-TSC algorithm in small- and large-scale regional road networks was verified.

### 4.3. Parameter Settings

The experimental parameters are delineated in Table 3; the models are trained for a total of 280 epochs. During the initial training phase, the experience replay buffer must accumulate a minimum of 300 samples before network updates can commence; the maximum buffer capacity is limited to 36,000 samples. The algorithm employs the Adam optimizer, with both the hybrid network and the independent Q-network sharing a learning rate of 0.001 and a discount factor of 0.99. During the training of the model, 32 sequences are sampled per batch, and updates are performed once per gradient calculation step. Gradient clipping with a threshold of 10 is applied to constrain the parameter updates of both the independent Q-network and the hybrid network, ensuring the stability of the coordinated optimization between global and individual strategies. The target network parameters are updated using a hard update method to synchronize with the main network parameters at intervals of 720 steps. The model is configured with a 4-head attention mechanism to enhance multi-agent collaboration capabilities.

The agent network employs an encoder–decoder architecture, comprising a multilayer perceptron (MLP) and a gated recurrent unit (GRU). The MLP contains two hidden layers, with 128 and 64 neurons, while the GRU’s hidden layer is set to a dimension of 64 to extract temporal features from local observations. The aggregation network is constructed on the basis of a multi-head attention mechanism, comprising a total of 4 attention heads, each with a dimension of 32. The generation of dynamic weights is derived from the global state $s_{t}$ via a lightweight network. During the training phase, the ε-greedy strategy is employed for the purpose of exploration. The initial value of ε is set to 1.0 and linearly decays to 0.05 over the first 50,000 training steps to balance exploration and exploitation.

Within the SUMO simulation platform, the duration of each training iteration is set to 3600 s, thus simulating one hour of traffic conditions within a real road network. In order to closely replicate actual traffic signal control scenarios, the agent’s decision interval is set to 10 s, which corresponds to the effective green duration of a signal phase. That is, every 10 s of green time, the optimal phase is reselected based on the currently observed state. The 3-s yellow interval, however, is automatically triggered by the environment upon phase termination and does not occupy any decision step (see Section 3.2.2). The experiments were implemented using the PyTorch deep learning framework (v2.1.0), and training and testing were conducted on an NVIDIA RTX 4060 GPU.

### 4.4. Experimental Results and Analysis

#### 4.4.1. Comparison Methods

In order to evaluate the performance of Qatten-TSC, a set of benchmark algorithms was selected for comparison, as listed in Table 4. The implementation of all algorithms was based on the states, actions, and rewards defined in Section 3.2.

The experiments serve two evaluation objectives. First, a parallel comparison with Fixed-Time, VDN, QMIX, WQMIX, QPLEX, and QPLEX-Attn is conducted to benchmark Qatten-TSC against representative value-factorization methods. Second, the effectiveness of our core innovation—the dynamic head-weighting mechanism—is validated via the ablation baseline Qatten-Base, which is the same as Qatten-TSC but with the state-dependent head-weighting replaced by equal-weight summation; the performance gap between the two therefore isolates the effect of the proposed mechanism.

Fixed-Time serves as the conventional benchmark for assessing the adaptive advantages of DRL methods over static signal plans. VDN is included to show the limits of linear decomposition in complex traffic networks, and QMIX to validate the advantage of nonlinear decomposition. WQMIX is selected because, like our method, it introduces weighting into value factorization but at the joint-action level via heuristic rules rather than on attention-head aggregation. QPLEX, one of the most expressive value-factorization frameworks, is included as a strong benchmark; its attention-augmented variant QPLEX-Attn further allows us to verify whether the proposed dynamic inter-head weighting offers genuine advantages over agent-wise attention-based credit assignment.

As summarized in Table 4, existing attention-based approaches aggregate multi-head outputs with equal weights (Qatten-Base), whereas Qatten-TSC conditions the inter-head weights on the global state, enabling credit assignment jointly across states, subspaces, and agents. Since the aggregation remains a linear combination of individual Q-values, the IGM property and linear complexity are preserved. It should be noted that this comparison also clarifies which design factors are controlled in our experiments: all DRL baselines have identical state, action, and reward definitions, which excludes richer state representations as a confounder. However, factors such as the number of attention heads, the decomposition of the state-dependency and the weighting itself within the head-weighting scheme, and parameter-capacity differences are not isolated in the current design; a systematic ablation covering these factors is left as future work.

#### 4.4.2. Small-Scale Road Network in Yangzhou

**Figure 8:**
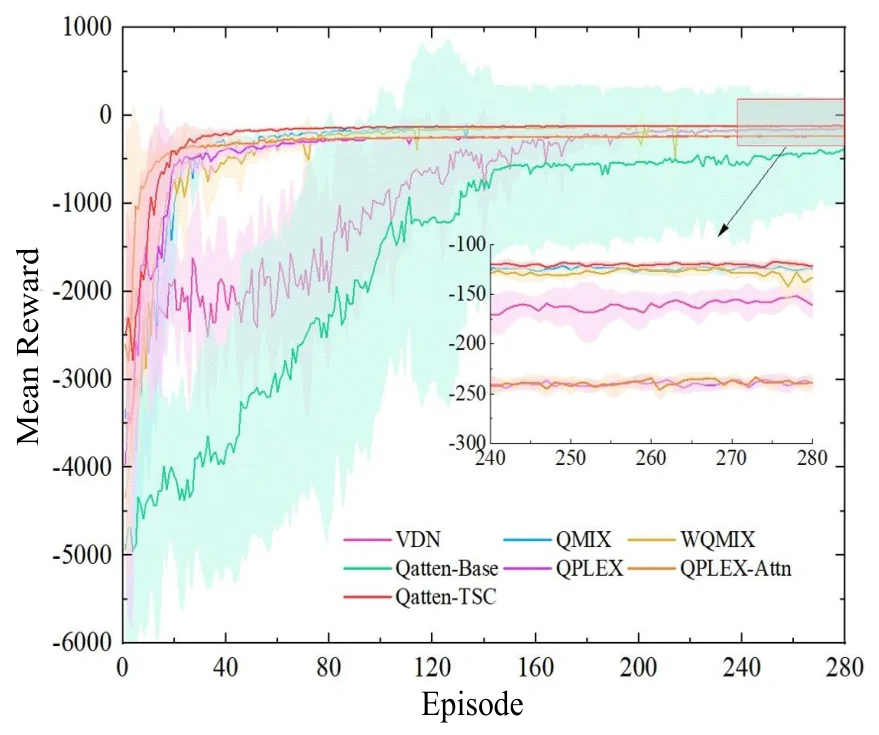
Comparison of Mean Reward in Small-Scale Road Network.

The objective of this study was to enhance the traffic efficiency of the small-scale road network in Yangzhou. The proposed methodology involved the minimization of queue lengths on all roads. The experimental results are presented in Figure 8. In the initial 30 training episodes, the enhanced Qatten-TSC algorithm attains reward values that are only surpassed by the QPLEX-Attn algorithm, which is recognized for its rapid convergence, and exhibits superior performance in comparison to the other reference algorithms. Following 30 training episodes, the Qatten-TSC algorithm exhibits superior convergence properties, with its reward values consistently outperforming those of competing algorithms, including QMIX and WQMIX. In contrast, VDN and Qatten-Base exhibit weaker convergence performance: the limitations of VDN are evident in its inadequate modeling of intersection interactions in its linear value decomposition. Qatten-Base, conversely, suffers from a weakening of key features due to the equal-weight fusion of multi-head attention. These shortcomings served to further validate the necessity of the dynamic weighting mechanism in Qatten-TSC.

**Figure 9:**
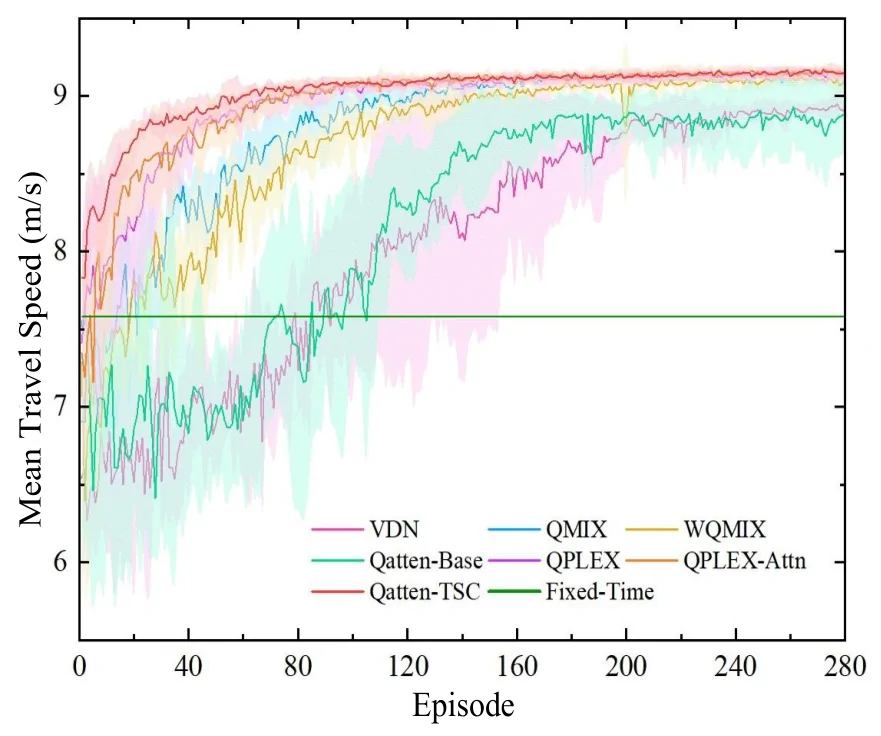
Comparison of Mean Travel Speed in Small-Scale Road Network.

As demonstrated in Figure 9, an investigation into the mean travel speed of disparate algorithms on the small-scale road network is provided. It is evident that the Qatten-TSC algorithm demonstrates the most rapid convergence during the initial 120 training episodes, consistently exhibiting superior average convergence rates in comparison to all deep reinforcement learning algorithms and traditional fixed-timing methods. Following 120 training iterations, Qatten-TSC, QPLEX, and QPLEX-Attn converged to similar stable states, with their average speeds after convergence being at the same level, indicating no statistically significant difference in performance. The WQMIX, Qatten-Base, VDN, QMIX, and Fixed-Time algorithms demonstrated substandard performance throughout the entire process.

**Figure 10:**
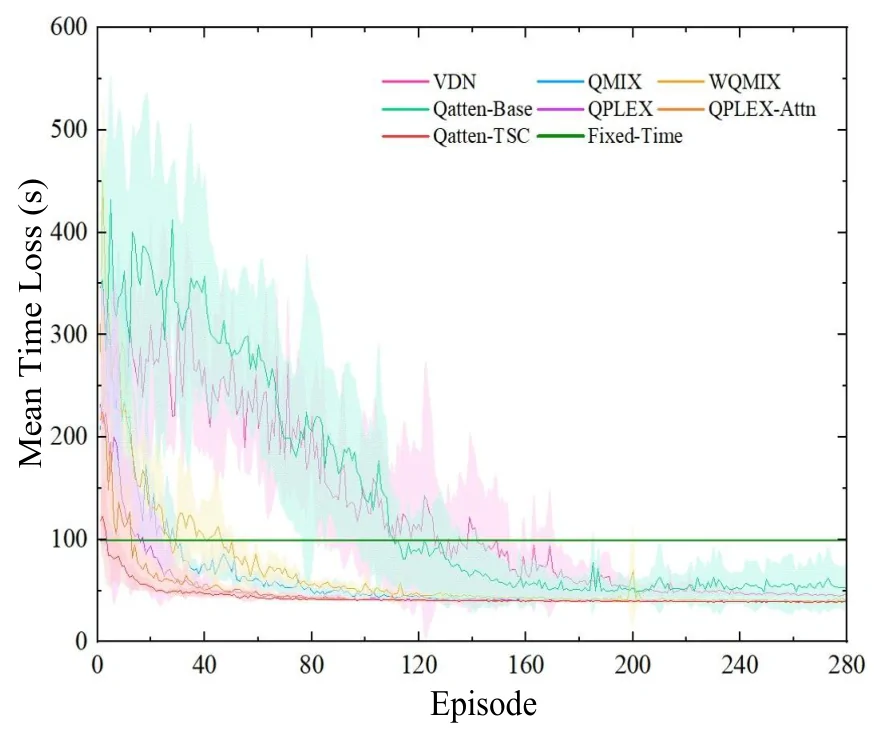
Comparison of Mean Time Loss in Small-Scale Road Network.

As demonstrated in Figure 10, the mean time loss metric demonstrates that QATTEN-TSC converges rapidly within the initial 80 rounds and minimizes the mean time loss, followed by QPLEX and QPLEX-Attn. Following 80 training episodes, all three algorithms demonstrate consistent superiority, significantly outperforming other algorithms such as QMIX. The experimental data demonstrated unequivocally that the enhanced Qatten-TSC algorithm exhibited strong performance in terms of both convergence speed and metric optimization.

In order to more intuitively analyze the traffic efficiency of various algorithms in the small-scale road network, the following statistical methods were employed: for each algorithm, the mean and standard deviation of the last 50 rounds after training convergence were calculated. It is important to note that within this time frame, the strategies employed by each algorithm had already reached a state of convergence. The standard deviation, both positive and negative, reflects the range of performance fluctuations in the post-convergence strategies, influenced by factors such as experience replay sampling and exploration noise. The resulting comparative metrics are displayed in Table 5. A comparison of the Qatten-TSC algorithm with other deep reinforcement learning algorithms, including QPLEX-Attn, QPLEX, VDN, WQMIX, and QMIX, reveals significant enhancements in terms of reward improvement. The Qatten-TSC algorithm demonstrates notable improvements of 49.69%, 49.93%, 26.4%, 6.34%, and 3.46%, respectively, signifying its efficacy in enhancing the performance of reinforcement learning systems. In terms of the mean time loss metric, Qatten-TSC reduces the time loss by 16.18%, 3.18%, and 1.72% compared to VDN, WQMIX, and QMIX, respectively, and performs roughly on par with QPLEX-Attn and QPLEX. With regard to mean travel speed metric, Qatten-TSC achieves improvements of 2.81%, 0.22%, and 0.44%, respectively, performing similarly to QPLEX-Attn and QPLEX. Moreover, the Qatten-Base algorithm, which integrates the contributions of attention heads, demonstrates the poorest performance across all metrics. In comparison with traditional fixed-timing methods, Qatten-TSC reduces mean time loss by 60.35% and increases mean speed by 20.56%. The metrics demonstrated the efficacy of the Qatten-TSC algorithm in enhancing overall traffic efficiency in the small-scale regional road network.

Qatten-Base demonstrated substandard performance across all metrics, with a reward of only −467.04 ± 709.96. Notably, its standard deviation is approximately two orders of magnitude higher than that of Qatten-TSC (±3.42). This suggests that the equally weighted sum of these variables can result in the interference of outputs from subspaces of varying importance, leading to significant fluctuations in Q-value estimates and making it challenging for the training process to converge stably. In contrast, Qatten-TSC employs head weights that can be adjusted, enabling the adaptive amplification of key subspaces and the suppression of redundant subspaces. This approach has been shown to enhance performance and stability. Concurrently, Qatten-Base took 38.9% longer than Qatten-TSC and was the slowest algorithm. The findings suggest that replacing the equally weighted sum with dynamic weighted aggregation enhances performance, validating the hypothesis that dynamic head weighting is the primary catalyst for performance gains in this method. The combination of the limitation of VDN being constrained by linear decomposition and the necessity of designing decomposition mechanisms tailored to the interactive structure of regional signal control tasks is further emphasized.

#### 4.4.3. Large-Scale Regional Road Network in Yangzhou

**Figure 11:**
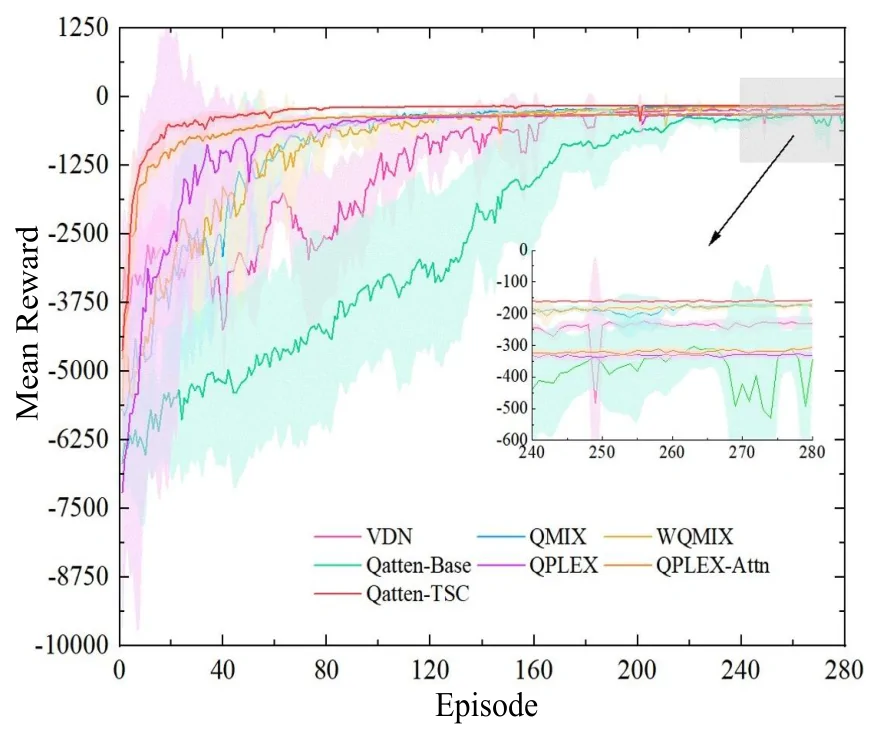
Comparison of Mean Reward in Large-Scale Regional Road Network.

The enhancement of traffic efficiency in the large-scale regional road network was realized through the minimization of queue lengths on all thoroughfares. The outcomes of the comparative evaluation are presented in Figure 11. As the scale and interaction complexity of the road network increase significantly, the Qatten-TSC algorithm, thanks to its ability to dynamically adjust the weights of individual attention heads, not only avoids performance degradation but also demonstrates better convergence and higher reward values, consistently outperforming all compared deep learning algorithms and traditional fixed-timing methods. Although the QPLEX-Attn algorithm initially demonstrates convergence second only to the Qatten-TSC algorithm, its reward values upon convergence in subsequent rounds are lower than those of the WQMIX, QMIX, and VDN algorithms. Conversely, algorithms such as Qatten-Base, which are incapable of effectively coordinating multiple agents, necessitate training for up to 220 rounds before gradually stabilizing as the complexity of the road network increased.

**Figure 12:**
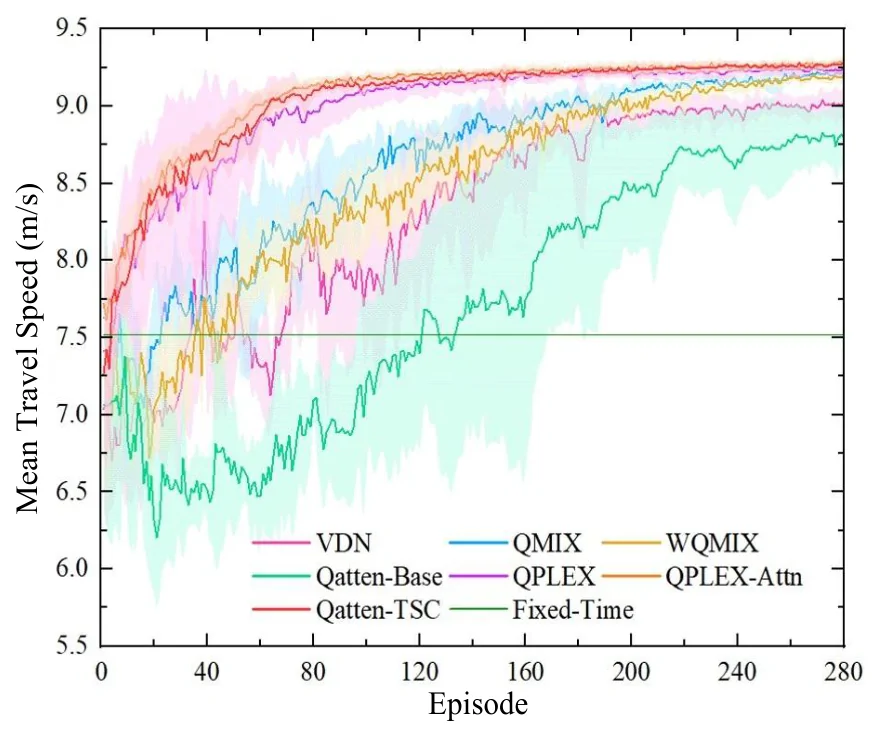
Comparison of Mean Travel Speed in Large-Scale Regional Road Network.

**Figure 13:**
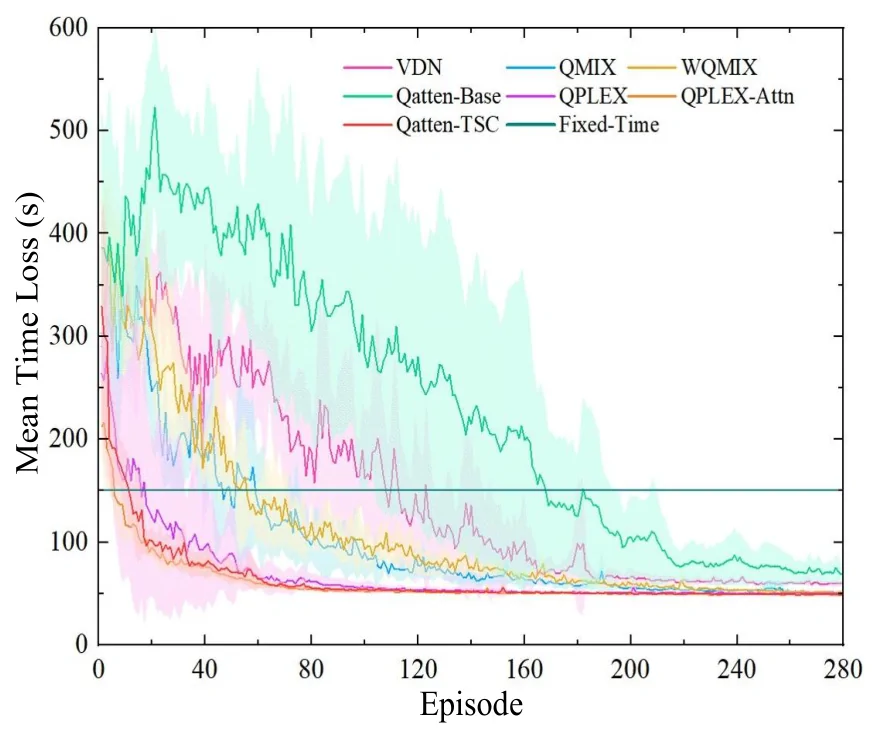
Comparison of Mean Time Loss in Large-Scale Regional Road Network.

Figure 12 and Figure 13 present a comparative analysis of the mean speeds and mean time loss of various algorithms in the large-scale regional road network. It is evident that Qatten-TSC and QPLEX-Attn exhibit not only rapid convergence and high average speeds but also excellent convergence stability and minimal time loss. The QPLEX algorithm demonstrates sub-optimal performance in comparison to the Qatten-TSC and QPLEX-Attn algorithms across both evaluation metrics. However, it exhibits superior performance in comparison to QMIX, WQMIX, VDN, Qatten-Base, and the Fixed-Time method. The Qatten-Base algorithm exhibited suboptimal performance due to its aggregation strategy, which involved directly summing the outputs of each attention head. This strategy led to inadequate representation capabilities in complex road network environments. The aforementioned observations indicated that the effective integration of the attention mechanism, achieved through dynamic weight allocation or optimization of credit allocation, was a viable approach to enhancing the adaptability of traffic control algorithms in complex scenarios.

In order to provide a more intuitive analysis of the performance metrics of various algorithms on the large-scale road network, the mean and standard deviation of the last 50 converged episodes for each algorithm were calculated, and then these two sets of data were averaged. The specific metrics are delineated in Table 6.

A comparison of the Qatten-TSC algorithm with other deep reinforcement learning algorithms, including QPLEX-Attn, QPLEX, VDN, WQMIX, and QMIX, reveals significant enhancements in terms of reward improvement. The Qatten-TSC algorithm demonstrates notable improvements of 49.78%, 51.76%, 34.12%, 12.78%, and 12.81%, respectively, signifying its efficacy in enhancing the performance of reinforcement learning systems. In terms of the mean time loss metric, Qatten-TSC achieves reductions of 19.28%, 6.52%, and 7.15% compared to VDN, WQMIX, and QMIX, respectively. It performs roughly on par with QPLEX-Attn and QPLEX. With regard to mean speed metrics, Qatten-TSC achieves improvements of 3.00%, 1.09%, and 0.87%, respectively, performing similarly to QPLEX-Attn and QPLEX. Concurrently, the Qatten-base algorithm, which calculates the contributions of attention heads, persists in demonstrating substandard performance across all metrics. In comparison with conventional fixed-timing methodologies, the Qatten-TSC algorithm was shown to reduce mean time loss by 70.80% and increase mean speed by 26.16% in the large-scale road network. These observations suggested that the algorithm was capable of enhancing traffic efficiency in the large-scale regional road network.

The experimental results support the necessity of head-level dynamic weighting in two ways: Firstly, Qatten-Base (without head-level dynamic weighting) demonstrated the poorest performance in both-scale road networks. Secondly, as illustrated in Figure 11, although QPLEX-Attn introduces inter-agent attention and rapidly converges in the initial stages, its rewards in the subsequent stages of large-scale road networks are lower than those of WQMIX, QMIX, and VDN. This suggests that inter-agent attention alone is inadequate for reliably estimating global value in complex road networks. The findings demonstrate that head-level dynamic weighting is an effective and necessary supplementary dimension to existing multi-attention value decomposition methods.

## 5. Discussion

The experimental results demonstrate that the Qatten-TSC algorithm exhibits superior performance in comparison to both traditional fixed-timing methods and existing MARL benchmark algorithms (VDN, QMIX, QPLEX) in both small- and large-scale road network scenarios. This finding suggests that traditional linear or static nonlinear value decomposition methods are unable to effectively capture the complex spatiotemporal dependencies inherent in regional traffic control. The performance disparity between Qatten-TSC and its ablation baseline, Qatten-Base, further substantiates the imperative for dynamic weighting, underscoring the observation that the contributions of disparate attention heads are not equivalent in dynamic traffic environments. Qatten-TSC’s state-dependent weighted aggregation has been demonstrated to adaptively highlight key interaction patterns, thereby effectively addressing the credit attribution problem in complex collaborative tasks. Furthermore, the reduction in average time loss and the increase in average speed indicate that this algorithm has significant practical value in real-world traffic management. Despite the encouraging results, it is important to note that there are some limitations that must be acknowledged. Firstly, it is important to note that the conclusions presented in this paper are based on post-convergence statistics from a single training run. Secondly, an 8-intersection network is inadequate for the rigorous validation of scalability in scenarios comprising thousands of agents. Thirdly, the Fixed-Time benchmark employs a synthetic, proportional four-phase signal timing scheme as opposed to real-world signal timing. Finally, conducting fully causal ablation experiments would further strengthen causal attribution. Such studies will be incorporated into future work.

## 6. Conclusions

The present paper addressed the complex dynamic interactions in regional traffic signal control by proposing a multi-agent reinforcement learning algorithm based on dynamic weighted value decomposition—Qatten-TSC. Specifically, a multi-head attention mechanism was integrated into the value decomposition network to capture spatiotemporal dependencies among intersections, and a dynamic weighted aggregation strategy was introduced to adaptively adjust the contribution of different attention heads based on the global state. Experiments on small- and large-scale SUMO simulation platforms based on real-world models of Yangzhou City showed the effectiveness of the proposed method. With regard to the key metrics of average reward, convergence rate, average speed, and average time loss, the Qatten-TSC algorithm was shown to outperform both traditional fixed-time methods and state-of-the-art algorithms such as VDN, QMIX, and QPLEX. Notably, the algorithm demonstrated strong adaptability, maintaining efficient control performance even as the complexity of the road network increased. In summary, the dynamic weighting mechanism proposed in this study successfully enhances the expressive power of value decomposition in collaborative tasks, providing a scalable and efficient solution for urban traffic signal control. 

## Figures and Tables

**Table 1:** OD Traffic Volume Table for Small-Scale Road Network in Yangzhou City.

Output	Input								
	1	2	3	4	5	6	7	8	Total
1	0	67	21	57	34	18	74	81	352
2	37	0	86	53	69	57	28	34	364
3	88	93	0	30	37	34	40	30	352
4	24	27	36	0	123	20	86	27	343
5	32	117	35	71	0	36	32	46	369
6	130	32	15	62	74	0	83	47	443
7	65	46	25	33	37	101	0	45	352
8	96	28	34	24	30	23	68	0	303

**Table 2:** OD Traffic Volume Table for Large-Scale Regional Road Network in Yangzhou City.

Output	Input												
	1	2	3	4	5	6	7	8	9	10	11	12	Total
1	0	34	25	29	14	30	5	44	38	20	72	20	331
2	64	0	16	33	27	27	28	14	9	18	28	20	284
3	28	57	0	10	27	43	20	30	29	21	32	9	306
4	24	17	82	0	23	10	26	17	32	25	37	7	300
5	22	21	35	63	0	31	32	26	23	10	25	13	301
6	26	20	52	32	77	0	14	27	33	20	30	8	339
7	12	11	23	0	47	21	0	15	8	16	9	18	180
8	36	7	14	24	20	23	59	0	35	10	32	25	285
9	20	33	15	29	28	27	27	91	0	10	24	12	316
10	19	27	30	13	34	29	15	12	62	0	18	10	269
11	19	16	25	13	24	15	22	12	21	58	0	10	235
12	18	8	13	18	21	12	17	17	19	52	18	0	213

**Table 3:** Experimental Parameter Settings.

Hyperparameters	Values	Hyperparameters	Values
Training Episodes	280	Target Network Update Steps	720
Learning Rate	0.001	Gradient Clipping	10
Batch Size	32	Number of Gradient Update Steps	1
Experience Replay Capacity	36,000	Minimum Threshold for Starting Training	300
Discount Factor	0.99	Number of Attention Mechanisms	4
Sequence Length	32	Optimizer	Adam

**Table 4:** A Comparison of the Mechanisms of Qatten-TSC and Existing Value Decomposition Algorithms.

Algorithm	Aggregation	Attention Placement	Credit Assignment Granularity	Key Difference
VDN [18]	Linear summation	None	Agent-level (static)	No subspace modeling
QMIX [19]	Monotonic mixing network	None	Agent-level (state-dependent)	No attention mechanism
WQMIX [20]	Weighted projection of monotonic factorization	None	Joint-action level (heuristic weights)	Weights not on subspace aggregation
QPLEX [21]	Duplex dueling decomposition	None	Agent-level	No attention mechanism
QPLEX-Attn	Duplex dueling decomposition	Individual advantage	Agent-level	Attention not on global aggregation
Qatten-Base [22]	Equal-weight head summation	Multi-head subspace	Agent-level	No adaptive inter-head weights
Qatten-TSC (ours)	State-dependent weighted head summation	Multi-head subspace	State × Subspace × Agent	-

**Table 5:** Specific Evaluation Metrics for Various Algorithms in Small-Scale Road Network.

Algorithm	Reward	Time Loss (s)	Speed (m/s)
VDN	−163.57 ± 18.13	46.92 ± 3.11	8.89 ± 0.07
QMIX	−124.71 ± 4.45	40.02 ± 0.74	9.12 ± 0.03
WQMIX	−128.55 ± 6.29	40.62 ± 1.04	9.10 ± 0.04
Qatten-Base	−467.04 ± 709.96	54.61 ± 22.77	8.84 ± 0.25
QPLEX	−240.45 ± 5.69	39.20 ± 0.57	9.15 ± 0.04
QPLEX-Attn	−239.47 ± 7.42	39.19 ± 0.66	9.15 ± 0.03
Qatten-TSC	−120.40 ± 3.42	39.33 ± 0.65	9.14 ± 0.04

**Table 6:** Specific Evaluation Metrics for Various Algorithms in Large-Scale Regional Road Network.

Algorithm	Reward	Time Loss (s)	Speed (m/s)
VDN	−243.64 ± 28.31	60.77 ± 2.82	8.99 ± 0.08
QMIX	−184.09 ± 17.46	52.83 ± 2.48	9.18 ± 0.04
WQMIX	−184.05 ± 10.93	52.47 ± 1.48	9.16 ± 0.03
Qatten-Base	−382.22 ± 156.59	75.19 ± 16.87	8.79 ± 0.25
QPLEX	−332.97 ± 12.80	50.03 ± 1.00	9.23 ± 0.03
QPLEX-Attn	−319.90 ± 9.58	49.04 ± 0.74	9.26 ± 0.04
Qatten-TSC	−160.51 ± 5.25	49.05 ± 0.87	9.26 ± 0.03

## Data Availability

Restrictions apply to the availability of these data. Data were obtained from Yangzhou Municipal Government and are available from the authors with the permission of Yangzhou Municipal Government.
